# Investigation of Automotive LiDAR Vision in Rain from Material and Optical Perspectives

**DOI:** 10.3390/s24102997

**Published:** 2024-05-09

**Authors:** Wing Yi Pao, Joshua Howorth, Long Li, Martin Agelin-Chaab, Langis Roy, Julian Knutzen, Alexis Baltazar-y-Jimenez, Klaus Muenker

**Affiliations:** 1 Faculty of Engineering and Applied Science, Ontario Tech University, 2000 Simcoe St N, Oshawa, ON L1G 0C5, Canadalangis.roy@ontariotechu.ca (L.R.); 2 Magna International, Aurora, ON L4G 7L6, Canada; 3 Magna Exteriors, Troy, MI 48098, USA; 4 Magna Exteriors, 63877 Sailauf, Germany; klaus.muenker@magna.com

**Keywords:** LiDAR, autonomous vehicle, rain, adverse weather, coating, surface wettability, optical analysis

## Abstract

With the emergence of autonomous functions in road vehicles, there has been increased use of Advanced Driver Assistance Systems comprising various sensors to perform automated tasks. Light Detection and Ranging (LiDAR) is one of the most important types of optical sensor, detecting the positions of obstacles by representing them as clusters of points in three-dimensional space. LiDAR performance degrades significantly when a vehicle is driving in the rain as raindrops adhere to the outer surface of the sensor assembly. Performance degradation behaviors include missing points and reduced reflectivity of the points. It was found that the extent of degradation is highly dependent on the interface material properties. This subsequently affects the shapes of the adherent droplets, causing different perturbations to the optical rays. A fundamental investigation is performed on the protective polycarbonate cover of a LiDAR assembly coated with four classes of material—hydrophilic, almost-hydrophobic, hydrophobic, and superhydrophobic. Water droplets are controllably dispensed onto the cover to quantify the signal alteration due to the different droplets of various sizes and shapes. To further understand the effects of droplet motion on LiDAR signals, sliding droplet conditions are simulated using numerical analysis. The results are validated with physical optical tests, using a 905 nm laser source and receiver to mimic the LiDAR detection mechanism. Comprehensive explanations of LiDAR performance degradation in rain are presented from both material and optical perspectives. These can aid component selection and the development of signal-enhancing strategies for the integration of LiDARs into vehicle designs to minimize the impact of rain.

## 1. Introduction

The development of intelligent features for road vehicles has been on the rise in recent years, with a focus on fully autonomous driving. There are five levels of autonomy as defined by the Society of Automotive Engineers (SAE). Level-0 offers no assistance, Level-1 offers adaptive features, Level-2 provides partial automation, Level-3 has conditional automation, Level-4 does not require driving intervention in preset environments, and Level-5 offers full automation in all conditions [1]. Common Advanced Driver Assistance System (ADAS) sensors include cameras, Light Detection and Ranging (LiDARs), Radio Detection and Ranging (RADARs), and Sound Navigation and Ranging (SONARs) [2]. The sensors are responsible for perceiving environmental data and communicating with ADAS when deploying automated functions, such as emergency braking, adaptive cruise control, hands-free steering, etc.

A concern regarding autonomous features is the uncertainty of sensor performance degradation when driving in adverse weather conditions, particularly rain, as it is the most common precipitation form globally. Optical sensors like cameras and LiDARs are more susceptible to impairments in the rain due to the use of shorter wavelengths (nm scale), which increase sensitivity to interference. Longer-wavelength (mid and high mm scale) sensors such as RADAR and SONAR are less prone to performance degradation as they have lower resolutions and their wavelengths are often larger than the raindrop scale (low mm scale) [3]. On the other hand, LiDAR is a less established technology than cameras, with multiple aspects still in development, especially for automotive applications, such as electronic and photonic components, signal processing, and packaging. Therefore, this paper focuses on investigating LiDAR performance in rain.

Automotive LiDARs typically utilize the time-of-flight principle to determine the range of objects based on simple elastic scattering phenomena without wavelength changes [4]. LiDAR emits laser pulses toward a target object; the detector then calculates the time it takes to receive a backscattered signal in order to identify the distance of the target from the incident source. A LiDAR sends out short-duration wave pulses with a known power, and the return flux is converted into an electric voltage using photodiodes or photomultipliers. The signal energy can be calculated with consideration of efficiency loss during the laser travel, as described in Equation (1) below. *E*, *σ*, *A*, *R*, and *η* represent energy, the cross-section of the target that is illuminated, area, range, and efficiency, respectively.

(1) $$E_{signal}=E_{transmitted}\cdot \frac{\sigma }{A_{illuminated}}\cdot \frac{A_{receiver}}{\pi R^{2}}\cdot \eta_{atmostpher}^{2}\cdot \eta_{system}$$

Automotive applications employ near-infrared lasers with a wavelength of 905 nm or 1550 nm, which are suitable for short-ranging. The energy of a 905 nm LiDAR is lower than that of a 1550 nm device for eye safety [5]. Lasers of 1550 nm wavelength may be sent out for shorter durations and by less affected by background noise; however, 1550 nm wavelengths are more prone to atmospheric absorption by gas molecules [6]. The current state-of-the-art types and sensing schemes of LiDARs are reviewed and discussed in [7] for the construction and operating mechanisms.

**Figure 1:**
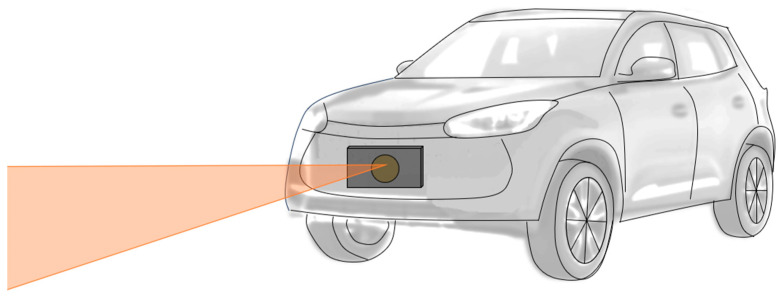
Demonstration of cover with integrated sensor modules behind the cover in a vehicle.

From a practical point of view, it is desirable to protect the ADAS sensors from harsh driving environments that may damage the sensor lenses due to soiling (surface contamination [8]). Thus, it has been proposed that protective covers may be used in front of the sensors, with various coatings applied to achieve properties such as water repellence, anti-abrasion, and anti-reflection [9]. Figure 1 shows the front cover, with integrated sensor modules behind.

**Figure 2:**
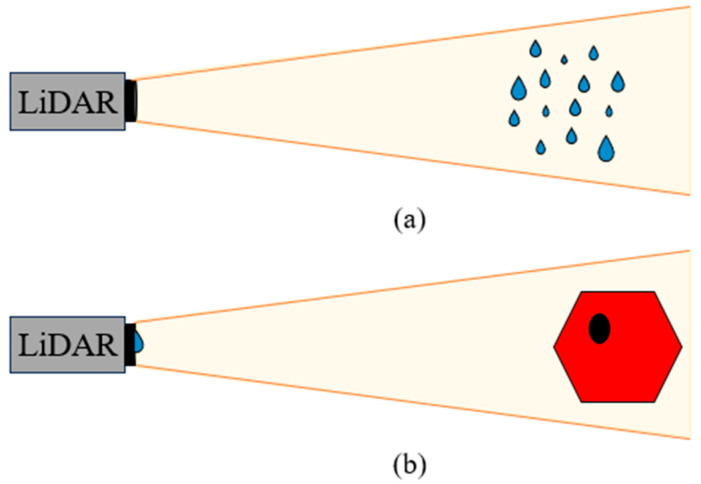
(**a**) LiDAR sees raindrops mid-air, which causes signal attenuation effects; (**b**) LiDAR sees raindrops adhering to the external surface of the sensor assembly, which causes signal deflection and obstruction effects.

Multiple studies suggested that LiDAR performance degrades in rain because raindrops can absorb laser energy or alter the paths of laser beams. The attenuation of light due to precipitation has been extensively studied concerning the extinction coefficients [5,10,11,12,13]; the experimental condition is demonstrated in Figure 2a, for which the adherence of droplets to the LiDAR surface is not considered. The attenuation effect on LiDAR return signal power *P_returned_* is shown in Equation (2), where *Z* is the distance of the object from the LiDAR. The extinction coefficient *ζ* is a function of natural rain intensity, drop size distribution, and the scattering effect caused by the droplets assuming spherical shapes. Also, assuming the only variable is rainfall rate, then the higher the rain intensity, the lower the return power.

(2) $$P_{returned}\propto e^{-2\xi Z}$$

Although the attenuation model provides an estimation of the LiDAR performance with respect to raindrop size distribution and intensity, there are other crucial factors to consider when driving in rain. Adherent raindrops are much closer to the LiDAR surface, causing obstruction and the deflection of signals [14]; this scenario is demonstrated in Figure 2b. The small apertures of the micro-electromechanical systems (MEMS) mirror, only a few millimeters in size, restrict the size of the scanning laser beam, which is one of the major factors obstructing LiDAR vision when raindrops of similar size to the aperture adhere to the outer surface of the LiDAR sensor [15]. The influence of aperture size is also discussed in [16,17]; generally, a larger aperture that can scan rapidly at a higher frequency is desired.

LiDAR performance degradation has also been studied for outer covers to prevent the addition of mechanical damage and surface contamination near the LiDAR aperture [18,19]. These studies showed that scratches on the cover affect the detection accuracy for a known position of the target. In contrast, extremely tiny droplets from dew can cause complete blindness and a reduced the number of points on the target when there are larger droplets present. Another recent study also reported missing measurements of LiDAR signals when exposed to rain [20]. These studies provided insights into the modes of detection faults based on several possible realistic scenarios, but did not investigate the causes of LiDAR performance degradation in depth. Understanding the problem fundamentally has a higher potential to allow the development of preventive measures, such as appropriate cover material and LiDAR optical component designs. 

With the evolution of ADAS sensors implementations, surface coatings are no longer only considered regarding overall body panel protection, but also the transmittance quality of sensor signals [21]. The size ratio between the contacting adherent raindrop and the aperture is dependent on surface material properties, which subsequently affect the raindrop, impacting characteristics and dynamics at the surface such as adhesion, motion, contacting area, and shape. Surface wettability is classified as hydrophilic, hydrophobic, and superhydrophobic when the static water contact angles (WCAs) are <90°, between 90–150°, and >150°, respectively. The resultant WCA, occurring due to surface roughness, is summarized in [22] with a review of electrodeposition coatings. The method used to evaluate surface wetting and droplet adhesion forces is summarized in [23] using a microbalance. Droplet impact dynamics are affected by surface wettability, resulting in different modes of motion, such as sliding, rolling, bouncing, splashing, and spreading [24,25,26]. Recently, there have been several works reporting the tuning of wettability gradients [27] and the patterning of hydrophobic/hydrophilic (biphilic) properties [28] to induce droplet movements; these could be interesting research directions in coatings for ADAS sensor applications and produce strategies with which to passively mitigate raindrops by facilitating water drainage. The droplet dynamics phenomena are expected to become more significant in driving scenarios with vehicle speed compared to being stationary.

**Figure 3:**
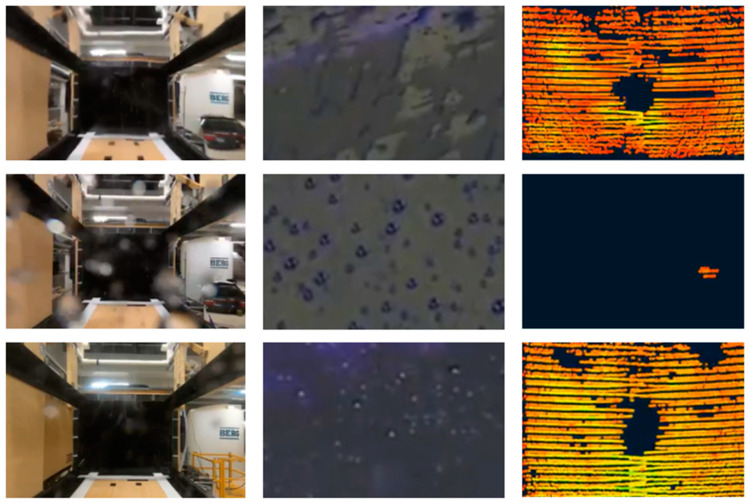
A matrix showing sample images of behind-cover camera perception, front-cover soiling behavior, and resulting LiDAR perception for hydrophilic (**top**), hydrophobic (**middle**), and superhydrophobic (**bottom**) covers. Soiling images are collected from wind tunnel rain tests based on the method reported in [29].

Our previous works show the surface material dependence during exposure to controlled and realistic simulated rain, as perceived by a moving vehicle [29,30], for which the use of different covers and coatings affects the optical sensor visibility of the detection target at a given condition. Sample images collected in simulated realistic rain via perception by a driving vehicle are shown in Figure 3 to demonstrate the significance of cover material in LiDAR performance; the controlled rain testing method, for which we used wind tunnels at the Automotive Centre of Excellence (ACE) at Ontario Tech University, Canada, is outlined in detail in [29,30].

Since LiDAR is an optical sensor, it is hypothesized that adherent raindrops of different sizes and shapes act as localized lenses for the LiDAR within its field of view (FOV), influencing optical paths. Some studies focus on the ball lens to achieve a beam coupling effect on optical paths [31,32], equivalent to the presence of an almost-spherical droplet on a superhydrophobic surface. Meanwhile, Ref. [16] discussed the ray-tracing model for an adherent hemispherical droplet, where the laser beam passes through a hydrophobic solid layer before reaching the droplet. They demonstrated that the ray deflection angle becomes more severe as the incident position becomes closer to the droplet curvature boundary. The droplet size factor is also investigated in their simulation and the results show that there exists an optimal ratio of droplet size to laser beam diameter for retaining a higher percentage of return laser power. A limited number of conditions were investigated in these studies. Therefore, the findings are not representative of a more comprehensive selection of cover materials but they provide insights for this study.

LiDAR performance degradation when driving in the rain is not very well understood in the field of automotive applications; therefore, it poses safety hazards when using autonomous features during rainy conditions. Currently, there are recommended requirements for automotive LiDAR systems [33] in terms of detection specifications, but there is no standard for the external materials used for automotive LiDAR applications, hindering the development of autonomous vehicles and the deployment of LiDAR in exterior applications. One of the main reasons for these research gaps is likely due to the lack of adherent droplet studies (droplets on cover), which are strongly related to the materials and optical properties of the surface. For instance, LiDAR performance for automotive applications, when driving, may not always follow the performance presented in typical studies in the open literature based on the return power estimation with respect to rain intensity resulting from attenuations due to droplets in the atmosphere, as Equation (2) does not incorporate factors such as driving speed, droplet size, and droplet dynamics arising from cover materials and vehicle aerodynamics. This paper is therefore motivated by the need to understand what causes LiDAR performance degradation in rain when in contact with adherent droplets. We hope that this will serve as a guideline for coating selection when producing automotive LiDAR covers that meet the application requirements of transmittance and weatherability.

The objective of the paper is to address the above-mentioned gaps by providing reasonings for LiDAR performance degradation by conducting a fundamental investigation of isolated droplet tests and discussing the results from materials and optical perspectives using four different classes of cover materials—hydrophilic, almost-hydrophobic, hydrophobic, and superhydrophobic. The focus is on the effects of frontal covers on LiDAR performance when droplets are present. 

The contributions of the paper are as follows:

- Comprehensive fundamental evaluation methodology, including optical and material aspects, for analyzing the suitability of a material to be used as a LiDAR cover;
- Wide range of materials are studied, spanning the full spectrum of wettability;
- Phenomenological models to explain the interactions between laser optics, droplet characteristics, and surface material properties;
- Baseline reference for modeling LiDAR performance in rain with respect to cover material properties;
- Criteria for enabling different types of materials to maintain LiDAR vision in rain;
- Research directions for LiDAR signal-enhancing strategies using material and optical approaches;
- Insights into the areas of soiling behavior and sensor responses for virtual ADAS sensors and AV simulation.

## 2. Materials and Methods

**Figure 4:**
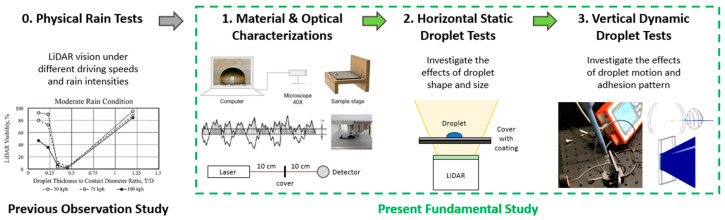
Overall approach for investigating LiDAR vision when driving in the rain.

The investigations of droplet influence on LiDAR sensor signals are categorized into three varieties: cover material and optical characterizations, horizontal cover orientation with static droplet and LiDAR facing upward, and vertical cover orientation with dynamic droplet using an optical test bench and numerical simulation. The overall investigation approach is explained in Figure 4.

### 2.1. Material and Optical Characterizations

Four classes of cover materials are used in this study—hydrophilic, almost-hydrophobic, hydrophobic, and superhydrophobic. The covers are made of LiDAR-grade tinted polycarbonate (optimized for 905 nm); the automotive application standard is met with recommended transmittance higher than 70% [34]. The wettability of the cover’s external surface is tailored by applying different automotive-grade silicone-based hard coatings, with recommended transmittance higher than 90% [35]; the superhydrophobic cover has an extra layer of commercially available low-surface-energy thin film, advertised for automotive sensor application on top of the hard coat.

The cover material’s properties are characterized under stable ambient conditions of wettability using a sessile method [36] goniometer stage and surface morphology is assessed by measuring white-light interference (WLI) on the Profilm 3D surface profilometer. On the other hand, optical properties are characterized regarding transmittance and reflectance is determined using a customized optical test bench.

The static water contact angle (WCA) is measured using a 10 μL water droplet dispensed by a micro-pipettor. The choice of droplet volume is within the typical measurement range [37], which is similar to the size of a natural raindrop [38], and it is controllable on a superhydrophobic surface to prevent motion. A 2D image is taken with a 40× camera lens facing towards the side of the droplet, and then the WCA is calculated using a tangential line technique with the open-source ImageJ software. On the other hand, surface morphology is a contributing factor to the WCA and optical efficiency. Light waves of different wavelengths are emitted towards the cover, focusing on an area of approximately 1 cm × 1 cm using a 20× lens; half of the beam is used as reference, while the other half is for measurement. Interference occurs when the reflected and reference beams collide, causing changes in the amplitude of the combined wave. The interference pattern is then interpreted by assessing the roughness the cover surface and measuring its peaks and troughs. For LiDAR application, cover arithmetic mean height (*Sa* [μm]) is the most relevant parameter for demonstrating the overall roughness profile to explain droplet behavior and optical properties. It is defined by Equation (3), which expresses the average height difference of each peak or trough compared to the surface arithmetical mean terms of in absolute value. Both WCA and surface roughness are averaged from 10 locations on the cover, with 5 locations along each longitudinal side, consistent with the cover manufacturing process.

(3) $$Sa=\frac{1}{A}\int_{A}\int \left|Z(x,y)\right|dxdy$$

An optical test bench is customized with an aluminum platform. It has a series of optical components to generate a laser beam of varying wavelengths and measure its total power. Specifically, an interchangeable 905 nm laser (Laserglow Technologies, LRD-0905 Series) is used in this study, which is aligned inside the test bench to pass through a removable flat cover at a perpendicular angle. A detector is positioned at an equal distance at the other end, sitting directly in the path of the laser to measure its power. The percentage of total power that is transmitted and reflected through the cover material is derived from the ideal situation without a cover over a range of angles of incidence between 0 and 70°. Prior to material and optical measurements, the cover sample is thoroughly cleaned with a soap solution and wiped with a microfiber cloth, except for the superhydrophobic cover, which is handled carefully by rinsing with water and shaking it dry.

### 2.2. Horizontal Static Droplet Tests

A high-resolution, pulsed-TOF, MEMS-based raster scanningvia an oscillating-mirror, forward-sensing 3D 905 nm LiDAR with linear angular resolutions and a FOV of about 70° × 20° (H × V), is used for the study; the laser source is assumed to be unpolarized. A LiDAR signal is set to capture the first reflection mode as it is likely to be more important for a vehicle to avoid the closest object rather than that with the strongest reflection one when navigating. The LiDAR unit is oriented facing upward, with a flat surface positioned 2.25 m from the optical center as a detection target. The LiDAR used in this study has a threshold of 10% reflectivity; points are filtered out if the signal is too weak, contributing to a small degree of uncertainty. The target detection analysis size is restricted to 0.5 m × 0.25 m of the surface, and the rest of the FOV is masked. Ten different volumes of droplets ranging from 0.5 to 30 μL are dispensed onto the cover within the 0.5 × 0.25 m custom FOV. A point cloud image is then taken, and the time gap between dispensing and taking the image is kept the same as that used during static WCA measurements to correlate droplet shape and LiDAR vision, as well as to minimize the variation in evaporation behavior.

Visibility is defined as the area of points present and reflectivity represents the averaged signal strengths received from the visible points. Visibility is measured by using image processing techniques on the point cloud images. The point cloud images are converted to 8-bit grayscale with the threshold set to make the image binary, and then the contours of points are mapped for area computation. Changes in visibility and reflectivity are quantified in two stages, the first comparing dry LiDAR performance on the effect of cover, and the second comparing LiDAR vision with the effect of a droplet on the cover. Assessing the number of points is another common method of quantifying LiDAR performance; it is usually employed for conditions that involve 3D targets, the purpose for which it was used in our other studies [29]. But for this fundamental study, the area method is more suitable as the ground truth is a 2D surface and evaluation of area blockage arising from the presence of a droplet is the primary interest.

### 2.3. Vertical Dynamic Droplet Tests

The effect of droplet sliding on the optical signal is first investigated using the same optical test bench setup with the introduction of a droplet that slides down naturally due to gravity. A single 10 μL droplet is dispensed onto the cover using a micro-pipettor at a position that is just above the intersection point of the laser. As the droplet begins to slide down the cover, the percentage of total power transmitted is measured with the detector in real time at 10 Hz. The procedure investigates the influence of the vertical length and curvature of a droplet on disturbances in laser transmission and the resultant transmitted power. Since the detector inside a LiDAR unit is typically positioned next to the transmitter, it is assumed that a large divergence in the laser path will result in the reflected signal not reaching the detector. Each case is repeated three times for hydrophilic and hydrophobic covers in order to obtain insights, as these are relatively controllable physical experiments compared to a non-wetting superhydrophobic cover that is only modeled numerically afterward.

While an optical test bench can provide insights into the behavior of a single laser beam in a LiDAR system in terms of signal power, it leaves questions unanswered, such as tracing the laser paths that are affected by a droplet. Therefore, the optical paths are simulated with COMSOL’s optical solver, which is used to further understand the phenomena. Hypothetical droplets and water films are modeled based on images taken from different angles during wind tunnel rain experiments using the method reported in [29]. Two scenarios are studied with respect to the physical experiments: (1) we simulate a single beam with droplet sliding by varying the height of the droplet while keeping the emitter and detector position constant; (2) we simulate LiDAR point cloud vision by having multiple beams and a 2D target. In this case, series of static droplets are modeled to visualize the resulting blockage behaviors. In addition to individual droplets, water film is also modeled as a film formation, which is common under high droplet-impact-velocity and high-rain-intensity conditions.

The droplets are modeled in freeform fashion based on physical measurements of droplets on the actual cover during our previous studies, such as in [29], similar to the ones shown in Figure 3; the critical values correspond to the droplet WCA, contact diameter, and thickness. While keeping the shape constant, the contact diameter and hence the droplet size was varied according to the ratios of droplet thickness to contact diameter (t/D) reported in [9]. Similarly, droplet positions were simulated by observing the trends on the cover soiling images in physical tests. A bi-directional coupled ray-tracing model is used in COMSOL to produce the point clouds. The model uses a refractive index in combination with Snell’s law to identify ray paths after passing between mediums, with a focus on only specular reflections. The refractive indices used for water and polycarbonate are 1.333 and 1.586, respectively. Fresnel’s equations [39] are then used to compute the intensity, which is the power of the transmitted ray. 

## 3. Results

As stated earlier, this paper aims to provide some insights into LiDAR performance degradation from material and optical perspectives by correlating characterized properties, varying droplet morphology, and tracing laser beams. At the end of the paper, phenomenological models are proposed for the four classes of cover materials—hydrophilic, almost-hydrophobic, hydrophobic, and superhydrophobic. In this section, the results are organized into four sub-sections: cover surface material properties, cover optical properties, LiDAR performance with the presence of droplets, and fundamental optical characteristics.

### 3.1. Cover Material Properties

**Figure 5:**
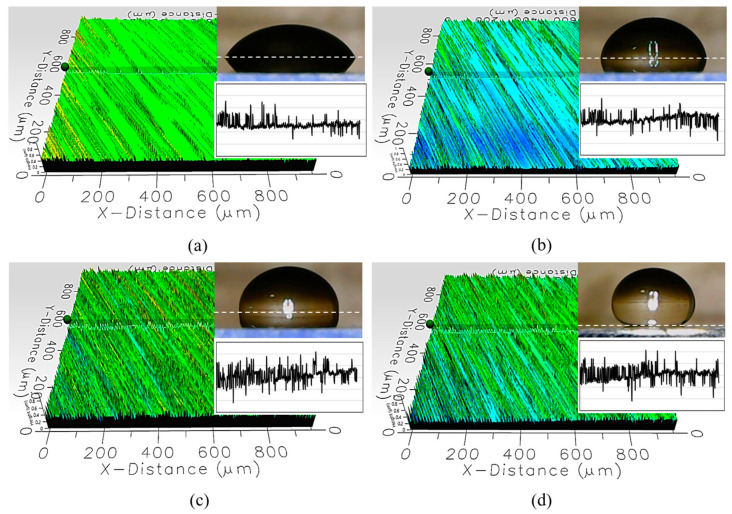
Qualitative demonstration of the material characterizations of the (**a**) hydrophilic; (**b**) almost-hydrophobic; (**c**) hydrophobic; and (**d**) superhydrophobic covers as determined via static water contact angle and surface roughness. Results are given at the same scale.

The cover material’s properties are comprehensively characterized via measurements at 10 different spots on the cover, assessing cover thickness, surface roughness, static WCA, and the ratio of droplet thickness to contact diameter (t/D). The visuals of these evaluations are shown in Figure 5. These properties are hypothesized to affect LiDAR performance; the average means and standard deviations from the 10 points of measurement are reported in Table 1.

Table 1 and Figure 5 suggest that the coatings are even, with small standard deviations in the thickness and roughness measured over 10 different spots. The superhydrophobic cover has a low-surface-energy thin film on top of the hard coating; hence, it is thicker by about 0.3 mm compared to the other covers. The hydrophobic coating is the roughest, which likely contributed to making it approximately 0.1 mm extra thick compared to the hydrophilic and the almost-hydrophobic covers, considering they underwent the same coating process.

Fewer peaks are observed for the hydrophilic cover, which is also reflected in the arithmetic mean roughness that it is the smoothest among the set of covers. Rougher textures with tighter gaps between the peaks are seen as the WCA increases across the set of four covers. However, with reference to the Wenzel model [40], the measured WCAs suggest that these four as-received cover coatings all have different chemical properties as they do not result in the same Young angles after applying their respective roughness factors. This is a crucial piece of information for understanding optical properties, as transmittance may be affected by material compositions in addition to thickness and surface roughness. Material compositions are not characterized in this study due to commercial restrictions.

WCAs describe surface wettability by classifying the overall droplet behaviors, such as droplet shape and spreading energy. To better understand the LiDAR performance from the optical perspective, wettability is quantified using a ratio of thickness to contact diameter (t/D) in addition to static WCA. The main reason is due to the hypothesis that droplets are small, localized lenses that change the optical path. Therefore, the curvature and contact profiles are key parameters dictating the optical paths with reference to Fresnel’s equations [39], explaining transmission, reflection, and refraction.

### 3.2. Cover Optical Properties

**Figure 6:**
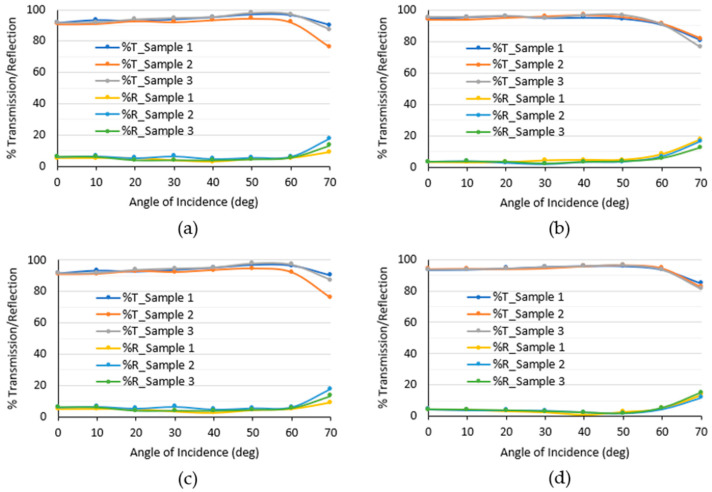
Transmittance and reflectance measurement for the (**a**) hydrophilic; (**b**) almost-hydrophobic; (**c**) hydrophobic; and (**d**) superhydrophobic covers using an optical test bench.

Cover optical properties are characterized by transmittance (%T) and reflectance (%R) over different angles of incidence and by measuring at three different spots on the cover, labeled Samples 1, 2, and 3 in Figure 6. As mentioned before, the cover is made of polycarbonate, with coatings applied to the external surface when used with a LiDAR unit. The laser path sequence is as follows: it leaves the laser source, traveling through the air; then, it meets the polycarbonate cover with coating on the outer side; and then it travels through the air again toward the detector.

The amount of incident power reflected changes depending on the polarization of light, whether it be unpolarized, s-polarized, or p-polarized. Using Fresnel’s equations, for p- and s-polarized lights, the total transmission decreases while reflectance increases as the angle of incidence increases between a light source and a medium. In this paper, p-polarized light is used; there is an anomaly that is presently known as the Brewsters effect, occurring specifically at an angle equal to the arctan of the refractive index of the second material (polycarbonate, *n* = 1.586) over the first material (air, *n* = 1). Ideally, there should be no reflection; however, in real applications, this leads to a higher transmission and lower reflection in some of the covers at around a 57.7° angle of incidence for a polycarbonate material.

The refractive index changes slightly with an added thin layer of coating, which causes differences in transmission and reflection for the four covers. The same idea can be applied for anti-reflection purposes by creating interference; the reduction in reflection will result in an increase in transmission through the material. In contrast, surface roughness may cause deviations in measurements as light is diffused away from the original position, which lowers the measured power. As mentioned in the description of the cover material properties above, the transmittance and reflectance are affected by both surface roughness and the material composition. Although the covers cannot be directly compared, the optical results presented provide a reference for functional LiDAR covers and the recommended placement of the cover with respect to the LiDAR lens to maximize transmittance and minimize reflectance in order to ensure good LiDAR vision.

### 3.3. LiDAR Performance with the Presence of Droplets

The LiDAR visibility attained when using a cover is compared to the baseline without a cover. Results are reported in Table 2, and visibility was measured to be 87.3%, 86.6%, 89.1%, and 98.8% with hydrophilic, almost-hydrophobic, hydrophobic, and superhydrophobic covers, respectively. Using a cover lowers the LiDAR visibility slightly, which aligns well with the optical property characterization. It is worth noting that laser beams emitted by the LiDAR diverge in different directions to obtain a wide FOV; thus, they are incident to the cover at different angles. As a result, the dry visibility with a flat cover parallel to the LiDAR lens is expected to differ from the optical properties characterized by a single-point laser. However, the visibility should lie in the range of transmittance measured.

**Figure 7:**
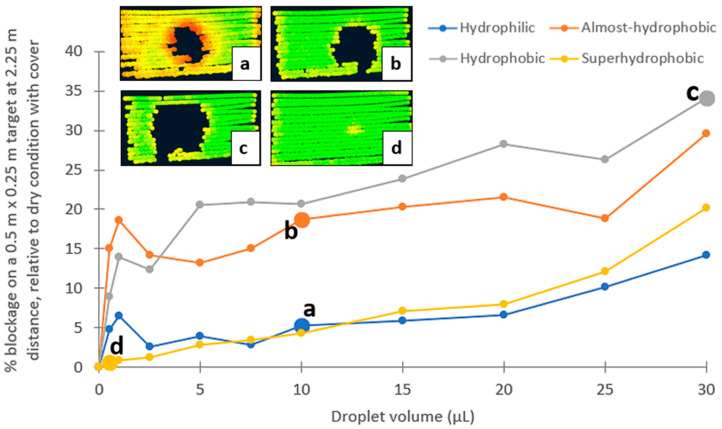
Percent missing point cloud (blockage) due to different droplet volumes on (**a**) hydrophilic; (**b**) almost-hydrophobic; (**c**) hydrophobic; and (**d**) superhydrophobic covers.

The influence of adherent droplets on LiDAR vision is investigated with controlled single-droplet tests with different droplet volumes. The presence of droplets causes missing points, which is referred to as signal blockage in Figure 7. The trend is non-linear; this could be because of several uncontrolled variables, including point cloud jittering, droplet molecular motion and spreading, droplet position, and the laser beams’ angle of incidence within the masked FOV region for accommodating different droplet volumes. In general, however, the larger the droplet, the larger the blockage and the lower the visibility.

The rate of increase in blockage size with respect to droplet size is faster with smaller droplets on hydrophilic, almost-hydrophobic, and hydrophobic covers. This may be caused by greater scattering, resulting in point clouds being less dense as partial beams are deflected. At smaller droplet sizes (≤2.5 μL), the blockage on the almost-hydrophobic cover is larger than that of the hydrophobic cover, likely due to the wider contact diameter from the spreading energy on the almost-hydrophobic cover and having a hemispherical shape. When the droplet volume increases (≥5 μL), the almost-hydrophobic cover induces more spreading and lowers the height of the droplet, which helps to retain more visibility than a hydrophobic cover.

From the selected sample images of the blockage behavior in Figure 7, it is observed that reflectivity (signal strength) is affected, as demonstrated by the change in color of the point clouds, with red having lower and green having higher reflectivity. When comparing the mean reflectivity in dry condition with the 30 μL droplet condition at points around the main blockage, a reduction in reflectivity is reported. This is recorder in Table 3, registering at 11%, 9%, 5%, and 8% for hydrophilic, almost-hydrophobic, hydrophobic, and superhydrophobic covers, respectively. The hydrophilic cover is most where the thinner region of the droplet is spread and resembles a water film, causing refraction and reduction in transmitted power. With less spreading, the almost-hydrophobic cover is 2% less affected than the hydrophilic cover. The hydrophobic cover generally causes missing point clouds without much effect on reflectivity changes; this is likely due to a consistent hemispherical shape regardless of droplet weight, such that either beams are being trapped or they are strongly deflected. The superhydrophobic cover case only performs well at smaller droplet volumes, and there are more partial droplet contacts due to droplet weight with larger volume, which begins to have a larger effect on the laser paths.

### 3.4. Fundamental Optical Studies

**Figure 8:**
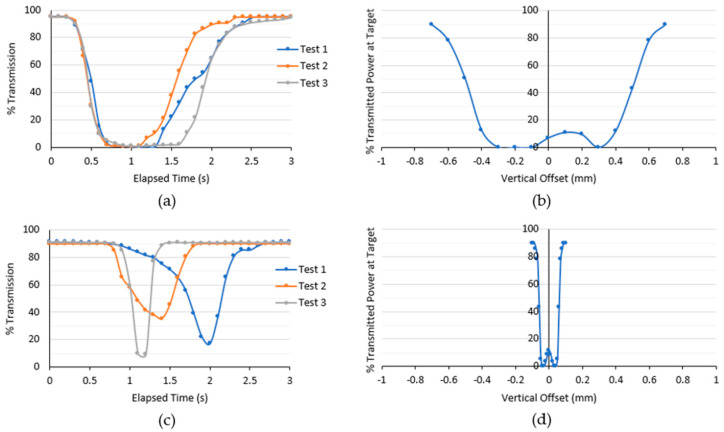
Influence on optical transmission due to a sliding droplet on a (**a**) hydrophilic; (**b**) almost-hydrophobic; (**c**) hydrophobic; and (**d**) superhydrophobic cover. (**a**,**c**) are recorded from physical experiments on duration of influence; (**b**,**d**) are recorded from simulation in COMSOL on the effect of position of the droplet.

To understand the phenomena of missing point clouds and the reduction in reflectivity due to the presence of a droplet on the cover, a droplet is dispensed physically, modeled on the cover, and allowed to slide down across the laser beam. It is found that the presence of droplets causes an obstruction to the laser signal as transmittance drops to almost zero for most cases. Figure 8a,c shows the duration of droplet influence on optical transmittance for hydrophilic and hydrophobic covers, whereas Figure 8b,d shows the effects of droplet position and curvature on optical transmittance for almost-hydrophobic and superhydrophobic covers without considering the sliding speed. A teardrop-shaped droplet slides down slower on a hydrophilic cover than to a hemispherical droplet on a hydrophobic cover, evidenced by the wider trough in the transmittance curve for the hydrophilic cover. This phenomenon aligns well with expectations as the hydrophobic surface is less wetting and can facilitate droplet removal faster. Meanwhile, an arbitrarily larger droplet (1.0 mm) on the almost-hydrophobic cover has a wider impact on transmittance than a smaller droplet (0.1 mm) on the superhydrophobic cover. The impact is quite symmetrical for a rounder droplet on the superhydrophobic cover. Some transmission is still permitted when the laser aligns with the center of the droplet, but its signal strength is weak. This is probably why the LiDAR point cloud shows the projected shadow of the droplet, with no point cloud seen at the center when this LiDAR unit has a 10% reflectivity threshold.

**Figure 9:**
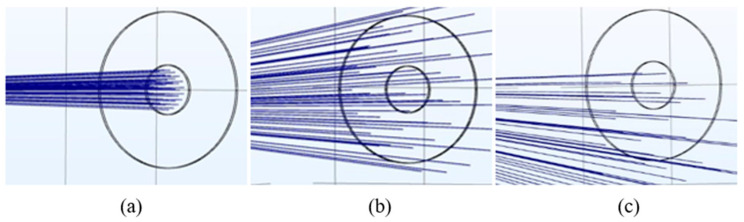
Laser paths for (**a**) no droplet; (**b**) a 1 mm hemispherical droplet with no position offset; and (**c**) a 1 mm hemispherical droplet with a 0.1 mm position offset.

Laser beams are traced in the simulation model to visualize the weakening of signal power that results in a decrease in optical transmittance when a droplet is in the laser’s path. Figure 9 shows the laser paths through a hydrophobic cover towards the detector in dry conditions and when a droplet is present at two different positions with respect to the center of the emitted laser beam. In a dry condition or one with no droplet (Figure 9a), most of the emitted laser power is captured by the detector on the other side, recorded to be 91% of the total power when having a 3 mm polycarbonate cover. However, when a 1 mm droplet is introduced (Figure 9b), the majority of the beam is deflected and does not reach the detector. Thus, the detected power is only 10% of the emitted power. Laser paths are observed to be sensitive to droplet position, which changes the angles of incidence at the droplet curvature. An offset of 0.1 mm (Figure 9c) of height to be lower than the center of the emitted laser beam further reduces the power by 5%. Therefore, it can be concluded that a droplet acts as a localized lens.

## 4. Discussion

### 4.1. Phenomenological Models

**Figure 10:**
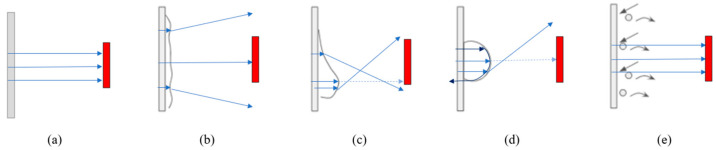
Phenomenological models explaining the behaviors of LiDAR laser beams for (**a**) dry condition; (**b**) hydrophilic cover with a disturbed film; (**c**) hydrophilic cover with a teardrop-shaped droplet; (**d**) almost-hydrophobic/hydrophobic cover with a hemispherical droplet; and (**e**) superhydrophobic cover with non-adhering droplets.

The goal of these fundamental material and optical studies is to be able to understand LiDAR vision when driving in the rain. By knowing the causes of performance degradation, better strategies can be developed to mitigate the soiling effects and enhance the signals. Phenomenological models are proposed for each class of material to explain what the soiling effects would mean to LiDAR performance when driving in the rain. Based on the combined effects on laser signals analyzed via the static droplet and dynamic droplet investigations, which correspond to the shape factor and time factor, respectively, the multiphase interactions are demonstrated in Figure 10. Overall, LiDAR performance degrades when adherent droplets are not removed quickly, and the direction of the originally emitted laser path changes such that fewer beams can reach the target object.

Droplets on a hydrophilic cover have smaller blockages in general, but they affect the laser signal for longer durations as they tend to slide down slower. A hydrophilic cover, such as the one used in this study with a WCA of 57°, typically forms teardrop-shaped droplets, such that there are regions of concave and convex curvatures (Figure 10c). The concave region causes laser beams to diverge, resulting in lower reflectivity at the top of the droplet. The convex region causes the convergence of laser beams with a large extent of refraction; this results in an extremely short focal length and the beams potentially not reaching the target. Therefore, point clouds become missing. For a more hydrophilic surface, e.g., glass with a WCA of <25°, or at higher driving speeds and stronger rain intensity conditions, droplets spread to form a water film. A stable water film is likely to act as another smooth layer of the medium that causes a slight reduction in signal strength as the laser beams are refracted to a small degree. The thicker the water film, the further the beams deviate from the target and the lower the detected power. This may also cause a shift in the detected object position compared to the ground truth. On the other hand, a disturbed water film has ripples that randomly deflect the laser beams in all directions (Figure 10b), resulting in a higher chance of having missing point clouds.

Both the almost-hydrophobic and hydrophobic covers behave similarly, but there is a small difference in droplet shape and dynamics arising from the 10° variance in WCA, which changes the droplet thickness to contact diameter (t/D) ratio on the cover. Droplets on the almost-hydrophobic cover have less curvature, which results in slightly smaller blockages. Both covers form almost-hemispherical droplets (Figure 10d), which cause the most severe LiDAR performance degradation condition. This can be explained by a combination of large refraction, a reflection of the laser beam near the sides of the droplet where incident beam angle is the shallowest, a weakening of the laser beam falling below the LiDAR detection threshold, and total internal reflection that traps the laser beam; all of these factors may lead to point cloud blockages. When driving in the rain, the cover experiences continuous droplet impacts; smaller droplets merge to form bigger ones and these begin to slide when the downward force exceeds the adhesion force. Normally, smaller droplets do not have enough weight to slide down quickly and tend to adhere to the spots where the large blockages remain. Therefore, LiDAR vision is poor with these covers unless droplet drainage can be facilitated to recover vision.

Superhydrophobic surfaces are known to be non-wetting; as such, droplets do not adhere to the cover. Droplets tend to bounce off upon impact with extremely short contact time, usually less than 20 ms [26], which is much faster than the LiDAR frame rate of ≤30 Hz [7]. From the optical studies, it is found that the laser beam only still travels relatively straight through the center of a curved droplet. Together with the non-wetting nature of the superhydrophobic cover, which only allows the adhesion of extremely small droplets, it also has the advantages of maintaining straight laser paths and LiDAR vision. 

### 4.2. Numerical Modeling of LiDAR Point Clouds

Numerical modeling is a time- and cost-efficient method of rapidly studying a wide range of conditions. It is also a beneficial tool that can accelerate the advancement of ADAS and autonomous vehicles (AV) by providing a visualization and optimization platform for LiDAR vision, with the presence of droplets of various sizes, shapes, concentrations, and locations. The simulated point clouds presented below can potentially revolutionize existing virtual ADAS/AV testing platforms with laser beam tracing capabilities and incorporate realistic rain influence on sensor performance due to the presence of adherent droplets on the covers.

**Figure 11:**
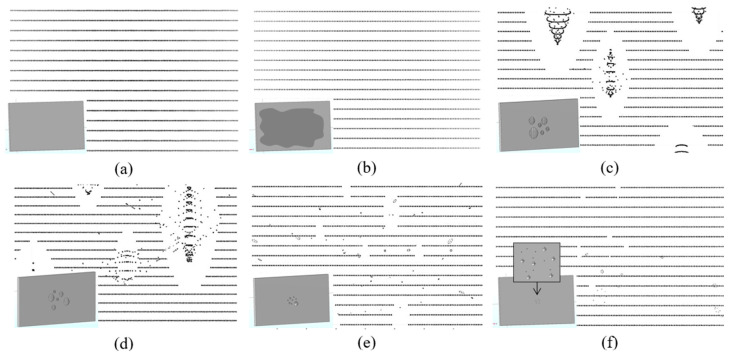
Simulated soiling condition and the resultant point clouds for (**a**) dry condition; (**b**) superhydrophilic cover with a stable film (WCA~5°); (**c**) more-hydrophilic cover with large spreading droplets (WCA~25°); (**d**) hydrophilic cover with large droplets (WCA~35°); (**e**) hydrophobic cover with hemispherical droplets (WCA = 90°); and (**f**) superhydrophobic cover with small droplets (WCA~150°). The modeled cover and droplets are shown at the bottom left for each condition.

Based on the observations of LiDAR signal blockages on the four different classes of cover materials—hydrophilic, almost-hydrophobic, hydrophobic, and superhydrophobic—the point cloud visual demonstration is extended to a superhydrophilic cover (WCA~5°) that forms a complete water film, and two more hydrophilic covers (WCA~25° and ~35°) that spread the droplets into a larger localized pool, shown in Figure 11.

The superhydrophilic cover (Figure 11b) retains full visibility, but the average signal strength over the entire target is weaker than in the dry condition (Figure 11a), as indicated by the lighter color of the point clouds. The two hydrophilic covers have blockages that wrap around the visible but distorted vision of the core region; this is validated by a physical experiment which shows that a larger pool of droplets results in lower reflectivity at the ring. This because there are curvatures at the edge of the droplet, while the center is relatively flat. The behaviors of the hydrophobic and superhydrophobic covers also align well with physical experiments where smaller droplets result in smaller blockages. The hydrophobic cover is seen to have some randomly distributed points; this is likely due to the reflections and refractions, causing a shortening of focal points where false detections occur at shorter distances compared to the ground truth. This behavior was also reported in a previous study [41].

### 4.3. Recommendations of LiDAR Signal-Enhancing Strategies

Through the comprehensive characterizations and fundamental investigations presented in this paper, several criteria for each class of cover material to maintain LiDAR vision in rain are derived from the findings, which they all revolve around droplet size, shape, and dynamics such as the post-impact motions. The criteria are presented in Table 4 in the form of desired extrinsic behaviors that must either operate via intrinsic characteristics by taking advantage of them, or working against them by inducing an unnatural behavior. Regardless of the materials used, the core aim is to either reduce the curvature of the droplets or to remove them quickly without multiplying the effects of these localized lenses. 

Signal-enhancing strategies can be grouped into four categories—material, optical, aerodynamic, and mechanical. Examples of each category are outlined below and are recommended for future exploration. In terms of the material approach, this study shows that both superhydrophobic and hydrophilic covers, considered as a single material, perform better than almost-hydrophobic and hydrophobic ones. However, hydrophilic covers only retain LiDAR vision in lower-rain-intensity conditions, as reported in our previous studies [9,29], and there are different material classes favoring certain driving and rain conditions. Patterning wettability or an electrowetting surface may be able to facilitate water drainage to prevent the formation of thick water films. On the optical approach, this study shows that droplets act as individual localized lenses that alter the laser paths. Therefore, compounding lenses is a potential solution to eliminate the curvature effect. However, it is a challenging task as the location of curvatures and the amount of water both need to be precisely controlled. On the aerodynamic approach, sensor cover surface morphology and orientation or adding air channels can be optimized to passively mitigate droplets; this method will become more effective as driving speed increases. On the other hand, active aerodynamic methods and mechanical approaches have already been commonly and extensively proposed, such as using blowers, wipers, fluid jets, and spray nozzles. To be effective, it may be beneficial to employ a hybrid approach, as each strategy has limitations and can usually only improve under limited conditions. 

## 5. Conclusions

This paper investigates automotive LiDAR performance degradation in rain from material and optical perspectives through fundamental studies using isolated droplets and various characterizations. The work demonstrates the state-of-the-art LiDAR-grade covers made of polycarbonate panel, which has good optical transmission in dry conditions at 905 nm. Four classes of cover coatings are used, including hydrophilic, almost-hydrophobic, hydrophobic, and superhydrophobic, the water contact angles (WCAs) are measured to be 57°, 82°, 90°, and 152°, respectively. The covers are further characterized by thickness, surface roughness, and the effect of angle of incidence on optical transmittance and reflectance to understand the property dependence of LiDAR vision.

When the LiDAR and cover assembly are exposed to a single droplet, it is observed that LiDAR performance degrades by missing point clouds or reducing signal strengths (reflectivity). The rankings of severity from the least affected to the most affected are superhydrophobic, hydrophilic, almost-hydrophobic, and hydrophobic. It seems counter-intuitive that a hydrophobic cover is an undesired approach to maintaining good LiDAR vision in rain, as there are a lot of commercially available hydrophobic coating products for soiling mitigation purposes. To explain this, the optical studies suggest that a hemispherical droplet formed on the hydrophobic cover has the highest chance of not having the laser beam reach the target object due to refraction and total internal reflection, thus, translating to missing point clouds. Raindrop influence on LiDAR point clouds is numerically modeled and expanded the demonstration onto a wider spectrum of wettability, including a superhydrophilic (WCA~5°) and two more hydrophilic (WCA~25° and ~35°) covers. This paper has focused on the investigation of laser paths during the complex multiphase interactions between air, water droplets, LiDAR cover, and the optical laser beam.

In general, LiDAR performance degrades when adherent droplets are not removed quickly, and the direction of the originally emitted laser path changes such that few beams can reach the target object, this includes both refractions and reflections caused by the adherent droplets acting as localized lenses. This is demonstrated in the phenomenological models proposed, which serve as signal-enhancing criteria. The material and optical analyses performed in this study provided insights into factors influencing LiDAR performance degradation. Based on the findings, several signal-enhancing strategies are recommended. The current work focuses primarily on the optical path and the amount of original laser power able to reach the target object. It provides critical insights for ADAS/AV operation in terms of visibility, i.e., the ability of the vehicle to detect an obstacle. However, in future work, the distance ranging should also be investigated as it is another critical aspect of sensor perception that determines the detection accuracy.

## Figures and Tables

**Table 1:** Quantitative characterization of material properties for the four classes of covers.

Cover	Cover Thickness	Arithmetic Mean Roughness	Static WCA	t/D Ratio with 15 μL Droplet
	(mm)	(nm)	(deg)	
Hydrophilic	2.90 ± 0.05	35.61 ± 4.42	57.08 ± 4.86	0.29 ± 0.03
Almost-hydrophobic	2.91 ± 0.03	40.05 ± 8.66	81.86 ± 2.06	0.41 ± 0.02
Hydrophobic	2.98 ± 0.01	52.31 ± 3.28	90.08 ± 0.44	0.45 ± 0.01
Superhydrophobic	3.21 ± 0.03	44.66 ± 5.42	151.95 ± 3.05	1.14 ± 0.10

**Table 2:** Measured LiDAR visibility in dry conditions using different covers in the sensor assembly, relative to a baseline without cover.

Cover	Visibility in Dry (%)
No cover	100.0
Hydrophilic	87.3
Almost-hydrophobic	86.6
Hydrophobic	89.1
Superhydrophobic	98.8

**Table 3:** Measured reduction in LiDAR reflectivity in the presence of a 30 μL droplet on the cover.

Cover	Reduction in Reflectivity (%)
Hydrophilic	11
Almost-hydrophobic	9
Hydrophobic	5
Superhydrophobic	8

**Table 4:** Criteria for different classes of cover material to maintain LiDAR vision in rain.

Cover	Resulting Droplet Size and Shape	Intrinsic Characteristics	Desired Extrinsic Behavior
Superhydrophilic	Large size, thin film	Complete wetting	Balanced in-flow and out-flow of water to maintain a slow-moving, stable film.
Hydrophilic	Large size, low curvature	Spreading	Shrink the droplets to reduce contact areas and remove them quickly.
Almost-hydrophobic	Medium size, large curvature	Unstable	Spread droplets through frontal forces on the cover.
Hydrophobic	Medium size, hemispherical	Slippery	Merge droplets to slide down with weight; fast removal without breaking into multiple smaller droplets.
Superhydrophobic	Small size, almost spherical	Non-wetting	Lower impact force for less penetration into the surface roughness to reduce droplet contact time.

## Data Availability

The original contributions presented in the study are included in the article, further inquiries can be directed to the corresponding author/s.
